# ISFET Biosensor with Loop-Mediated Isothermal Amplification for Electronic Rapid Detection of Mycoplasma Pneumoniae

**DOI:** 10.3390/s25051562

**Published:** 2025-03-04

**Authors:** Jie Zou, Jie Hu, Yan Shen, Limei Zhang, Weiyi Bai, Lei Wang, Jianlong Li, Lin Yan, Zhifeng Zhang, Hao Bai, Wenchuang Hu

**Affiliations:** 1 Precision Medicine Translational Research Center (PMTRC), West China Hospital, Sichuan University, Chengdu 610041, China; 2 Department of Laboratory Medicine, Clinical Laboratory Medicine Research Center, West China Hospital, Sichuan University, Chengdu 610041, China; 3 One-Chip Biotechnology Co., Ltd., Chengdu 610041, China

**Keywords:** mycoplasma pneumoniae, ISFET, CMOS, pH-LAMP, point of care

## Abstract

Mycoplasma pneumoniae (MP) is the main culprit of community-acquired pneumonia. Commonly used laboratory testing methods have many shortcomings. Serological diagnosis has low sensitivity, causing false negatives, while a quantitative real-time polymerase chain reaction (qPCR) requires large equipment and professional staff. To make up for these shortcomings, we proposed a label-free, low-cost, and small-sized ion-sensitive field-effect transistor (ISFET) array based on a low-buffered loop-mediated isothermal amplification (LAMP) assay. A complementary metal oxide semiconductor (CMOS)-based ISFET array with 512 × 512 sensors was used in this system, which responds specifically to H^+^ with a sensitivity of 365.7 mV/pH. For on-chip amplification, a low-buffered LAMP system designed for the conserved sequences of two genes, *CARDS* and *gyrB*, was applied. The rapid release of large amounts of H^+^ in the low-buffered LAMP solution led to a speedy increase in electrical signals captured by the ISFET array, eliminating the need for a sophisticated temperature cycling and optical system. The on-chip results showed that the device can accurately complete MP detection with a detection limit of about 10^3^ copies/mL (approximately 1 copy per reaction). In the final clinical validation, the detection results of eight throat swab samples using the ISFET sensors were fully consistent with the clinical laboratory diagnostic outcomes, confirming the accuracy and reliability of the ISFET sensors for use in clinical settings. And the entire process from sample lysis to result interpretation takes about 60 min. This platform has potential to be used for the point-of-care testing (POCT) of pathogen infections, providing a basis for the timely adjustment of diagnosis and treatment plans.

## 1. Introduction

Mycoplasma pneumoniae (MP) is one of the most common pathogens causing community-acquired pneumonia in children and adolescents [1,2,3,4]. MP infection has been on the rise recently and is an important factor in children’s respiratory health because of the increasing resistance to macrolide antibiotics in MP-infected individuals [5,6]. MP infections present mainly with cough and recurrent fever, similar to infections with other respiratory pathogens. Therefore, the clinical symptoms are not diagnostically specific [7].

The rapid detection of pathogens is considered an important tool for the early screening and prevention of pathogen transmission [8]. Commonly used diagnostic methods include serological diagnosis and quantitative real-time polymerase chain reaction (qPCR). The confirmation of MP infection in some centers is based on paired comparisons of acute and recovery serum samples, but the presence of a window period of infection may lead to missed tests, and serological diagnosis is less sensitive and time-consuming. qPCR has become the gold standard for the diagnosis of most respiratory pathogen infections due to its high sensitivity and specificity [9,10,11]. However, laboratory PCR has shortcomings in its need for bulky optical instruments and specialized laboratories and personnel, as well as its long testing time [12], limiting its application in outpatient clinics, emergency clinics, and grassroots hospitals. So, it is hard to provide doctors with the necessary basis for targeted treatment on the spot [13,14], which may lead to the inappropriate use of antibiotics and the continued spread of MP. Therefore, the development of a low-cost, portable, rapid, sensitive, and specific MP detection device is of great significance for the timely and accurate detection or exclusion of MP infections and the formulation of accurate clinical plans.

Unlike optical detection methods such as qPCR, electrochemical detection has the advantages of being label-free and low-cost, having a short testing time, and using miniaturized devices [15,16]. Among electrochemical detection devices, ion-sensitive field-effect transistors (ISFETs) can convert the concentration of target ions into output voltage signals (*V_out_*). With the varying concentration of target ions, *V_out_* changes accordingly. Previously, researchers built a variety of biosensors based on ISFETs, which successfully enabled the detection of nucleic acids [17,18,19]. But discrete ISFETs have limited reliability and multi-complexity. The use of complementary metal oxide semiconductor (CMOS) technology to fabricate ISFETs enables the formation of large sensor arrays and on-chip integration with circuitry [20]. These CMOS-based ISFET chips, with a high degree of integration and miniaturization, large multi-complexity, and low cost with volume manufacturing, enable applications in nucleic acid detection and gene sequencing [21,22].

In addition to PCR, loop-mediated isothermal amplification (LAMP), one of the most used isothermal amplification technologies, can amplify target nucleic acids exponentially at a constant temperature. There is no need for sophisticated temperature control modules like those required in PCR. Moreover, it was reported in our previous studies that the amplification of nucleic acids can cause a pH decrease in low-buffer LAMP systems, enabling the possibility of the label-free electronic detection of nucleic acids by pH-mediated colorimetric assays or pH sensors such as ISFETs [23,24].

**Figure 1:**
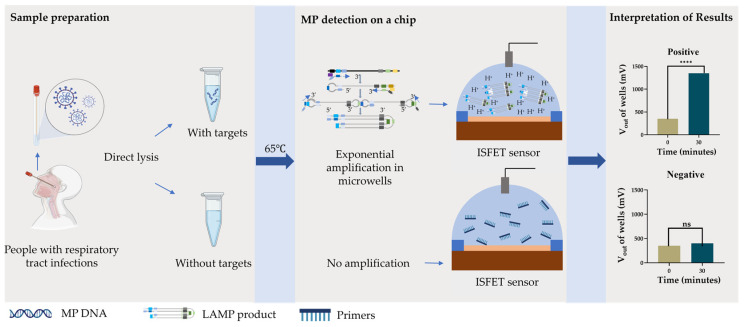
Workflow for MP pathogen detection with throat swabs of patients using ISFET biosensor integrated with pH-based label-free LAMP system. **** indicates *p* < 0.0001.

Here, we report an MP pathogen detection device combining a bio-chip of an ISFET array and the pH-based label-free LAMP technique for rapid, accurate, portable, and low-cost testing, as shown in Figure 1. This device integrates a bio-chip of 512 × 512 ISFET sensors with a LAMP reaction chamber, a temperature control module, and a signal readout circuit. This device can accurately detect MP targets in about 60 min from sample to answer with a sensitivity of up to 10^3^ copies/mL (approximately 1 copy per reaction). It has the potential to provide the timely diagnosis of MP-infected patients in outpatient clinics, emergency clinics, and resource-limited areas.

## 2. Materials and Methods

### 2.1. LAMP Primer Design and Conservation Analysis

The sequences of the MP *CARDS*, *gyrB*, and *SDC1* genes were downloaded from NCBI’s Nucleotide database. The conservation of these sequences was analyzed by using the MUSCLE algorithm and extracted at different cut-off values (99%, 95%, 90%) separately. LAMP primers targeting the conserved sequences of these genes were designed using LAMP designer v1.16. Experimental selection was performed using a fluorescent method to ensure amplification efficiency and specificity. 

### 2.2. LAMP Reaction Conditions

In the establishment of the LAMP reaction system, the concentrations of MgSO_4_ and KCl were optimized. The final ingredients are as follows: Each 25 μL experiment contained 1.25 μL Tris-HCl (1000 mM stock), 0.15 μL MgSO_4_ (1000 mM stock), 0.13 μL Tween-20 (20% stock), 1.4 μL dNTPs (25 mM stock of each nucleotide), 0.25 μL Bst3.0 (32 U/mL stock, HaiGene), 2.5 μL EvaGreen (20× stock, maokangbio), 2.5 μL 10× LAMP primer (4 mM F3 and B3, 32 mM FIP and BIP, and 8 mM LF and LB), and 2.5 μL template, and the remaining solution was topped up to 25 μL with nuclease-free water. The reaction was carried out at 65 °C for 40 min, followed by one melting curve analysis from 65 to 95 °C at a ramp of 0.5 °C/s to confirm the specificity of amplification products. Reactions were performed on a LineGene 9600 Plus (Bioer Technology, Hangzhou, China). All primers used in this study were purchased from Sangon Biotechnology Co., Ltd. (Shanghai, China).

For pH-based LAMP to achieve high amplification signals, we reduced the buffer capability of the LAMP reagent system. In this low-buffered LAMP, the component of Tris-HCl was reduced to 0.08 μL (1000 mM stock) in each reaction. Additionally, 0.05 μL KOH (1000 mM stock) was added to adjust the initial pH for the best amplification efficiency. Phenol red was used as the pH indicator for a comparative study and quality control during assay development.

### 2.3. Analytical Performance of Low-Buffered LAMP

The detection sensitivity of the low-buffered LAMP reaction system was evaluated by detecting the 10-fold concentration gradient dilutions of synthetic DNA samples ranging from 1 × 10^5^ to 1 × 10^3^ copies/mL. The time to positive (TTP) of each reaction group was recorded. To determine whether cross-reactions occurred and verify the detection specificity of this system, the established LAMP system was used to detect respiratory pathogens including influenza A virus (FluA), influenza B virus (FluB), respiratory syncytial virus (RSV), and adenovirus (Ad) with 10^5^ copies/mL concentrations.

### 2.4. Gel Electrophoresis

Gel electrophoresis was performed to verify the amplification products. First, polyacrylamide gel was prepared with 4 mL nuclease-free water, 2 mL 30% acrylamide, 120 μL 1× TAE buffer, 20 μL 10% ammonium persulfate, and 10 μL TEMED. Then, 2 μL DNA marker (25–500 bp, Sangon Biotechnology Co., Ltd.) was added, and the LAMP product was mixed with loading buffer (6×, Tsingke, Beijing, China) and then loaded into the precast wells of the gel. Next, the power supply was set to 100 V, and the experiment was run for 1 h. Finally, the results were interpreted using a gel imaging system (Bio-rad ChemiDoc Go, Hercules, CA, USA).

### 2.5. Fabrication and Working Principle of ISFET Sensors

**Figure 2:**
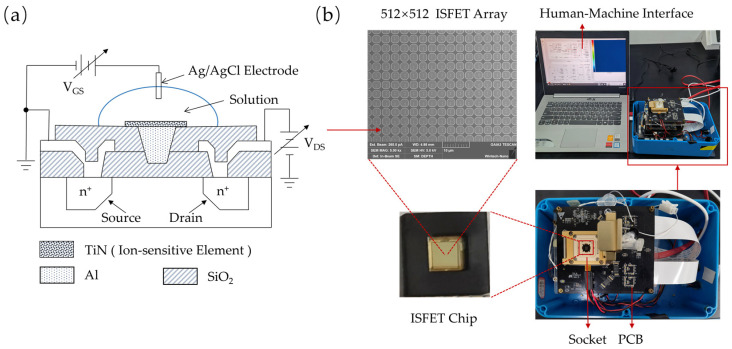
A schematic diagram of the structure of the ISFET-based H^+^ sensing platform. (**a**) The basic structure of the ISFET sensor. (**b**) The experimental setup for the H^+^ sensing platform.

The ISFET was designed and fabricated using commercial 180 nm CMOS technology (SMIC, Shanghai, China). Figure 2a shows a schematic diagram of an ISFET, where TiN is used as the hydrogen-sensitive material and AgCl/Ag as the reference electrode inserted in an electrolyte covering the ISFET.

The ISFET sensor operates by detecting changes in surface charges or potential that arise from the adsorption or desorption of hydrogen ions (*H*^+^) on the hydroxyl groups (−*OH*) of the TiN (titanium nitride) sensing membrane. Based on semiconductor theory and the derivation in [25,26], when the pH of the electrolyte changes, the protonation or deprotonation of these hydroxyl groups alters the surface charge density on the TiN membrane. This change in surface charge modulates the electric field at the membrane–electrolyte interface, leading to a shift in the threshold voltage (*Vth*) of the ISFET. The resulting variation in the drain–source current (*I_DS_*) or voltage (*V_DS_*) is then measured, providing a direct correlation to the pH of the electrolyte. Equation (1) shows that the change in drain–source Δ*V_DS_* is linearly related to the variation in the pH (Δ*pH)* of the electrolyte when the device operates in the linear region [27]:

(1) $$\Delta V_{DS}=G\cdot 2.303\frac{kT}{q}\alpha \;\Delta pH$$

where *G* is the signal amplification factor of the ISFET circuit, which is about 6× in our design. *α* is the ISFET intrinsic sensitivity factor between 0 and 1, as given by the following [27]:

(2) $$\alpha =\frac{1}{1+\frac{2.302kT\cdot C_{DL}}{q^{2}\cdot \beta_{int}}}$$

where *β_int_* and *C_DL_* are the buffer capacity and the electronic double-layer capacitance of the ISFET sensing surface, respectively. Specifically, the Nernst equation predicts that the theoretical sensitivity of an ISFET sensor at ambient temperature is about 2.302 *kT*/*q* = 59.5 mV/decade, where *α* is 1. This linear dependence highlights the ISFET’s capability to accurately detect and quantify pH variations in the electrolyte, making it a robust tool for pH sensing applications.

During the LAMP amplification reaction, one *H*^+^ is released for each nucleotide added, as shown in Equation (3):

(3) $$ndNTP+{DNA}_{m}\to {DNA}_{m+n}+{nPP}_{i}+nH^{+}$$

where *n* is the total number of nucleotides incorporated during amplification, so the change in *H*^+^ is proportional to the total number of nucleotides inserted. In low-buffer systems, a large amount of *H*^+^ generated by nucleic acid amplification accumulates on the TiN surface, causing a positive change in the surface potential and the resulting change in the drain current Δ*I_D_* and finally the output voltage *V_out_*. By this means, the ISFET sensors are able to monitor the progress of nucleic acid amplification reactions, and the combination of ISFET sensors and low-buffered LAMP assay can be used for a highly sensitive, label-free, and specific detection of MP.

### 2.6. ISFET-Based H^+^ Sensing System

The diagnostic device consists of an ISFET chip, signal acquisition module, temperature control module, and human–machine interface (HMI), as shown in Figure 2b. The ISFET array on the detection chip is composed of 512 × 512 ISFET pixels (262,144 sensors) with a TiN sensing pad size of 3 µm × 3 µm for each ISFET. Our readout circuit consists of two units. The first unit is a heating and temperature control module with pulse-width modulation (PWM) with close-loop constant temperature control. The second component is an embedded system designed to generate control signals and collect output data. It includes an amplifier module to convert current signals into voltage signals and an ADC module to digitize the analog voltage into digital signals. A custom socket was developed to connect the chip to the circuit system and the electrolyte solution. The ISFET gate voltage is applied through a reference electrode inserted into the solution.

Before the LAMP experiment, the ISFET sensor chip was wire-bonded on a PCB board and sealed with a cap. Then, a 1 μL reaction reagent was introduced through tubing above the gasket using a pipette and sealed with a drop of paraffin oil, forming a thermostatic amplification chamber. This design effectively prevented the evaporation and contamination of amplification products. Voltage signals were acquired by transmitting *V_out_* to a computer via an analog-to-digital converter circuit, with a Ag/AgCl electrode providing the required gate voltage. The LAMP reaction was initiated by heating the sensor to 65 °C using an integrated temperature control module, maintaining this condition for 30 min. During the LAMP, hydrogen ions generated by amplification interacted with the hydroxyl groups of the TiN sensing membrane via selective surface protonation reactions, causing a change in the surface charge and the sensor’s *V_out_*. After the reaction, the temperature was reduced to 25 °C for signal acquisition. Finally, the average of the voltage outputs of all ISFET sensors before and after amplification was recorded, and the resulting change was used to evaluate the assay results. Negative controls using nuclease-free water were used to ensure experimental reliability.

### 2.7. Detection of Clinical Samples

To evaluate the analytical capability of the ISFET sensing platform for MP, we collected 8 throat swab samples from West China Hospital of Sichuan University. Throat swab samples were divided into two aliquots. The first aliquot was used for direct lysis in lysis solution containing 0.5% Triton, 75 mM KCl, and 2 mM KOH. And then detection was conducted by the ISFET sensing platform. The other portion was used for clinical laboratory diagnosis. The samples were subjected to nucleic acid extraction and purification using a commercial nucleic acid extraction kit (Sansure Biotech, Changsha, China), followed by PCR amplification and the capillary electrophoresis of the PCR products (Healthgenetech, Ningbo, China). A peak value greater than 1000 was interpreted as positive in the electropherogram. The results were then compared with the detection outcomes from the ISFET sensing device.

## 3. Results

### 3.1. Performance of ISFET-Based Sensing Platform

**Figure 3:**
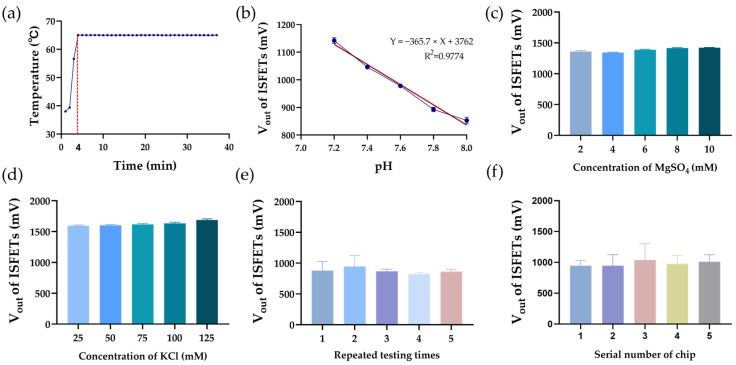
Performance of ISFET-based sensing platform. (**a**) Temperature of detection chip over time; (**b**) *V_out_* of PBS solutions with different pH; (**c**) *V_out_* of PBS solutions with gradient concentrations of MgSO_4_; (**d**) *V_out_* of PBS solutions with gradient concentrations of KCl; (**e**) *V_out_* of 5 repeated testing on same chip; (**f**) reproducibility of *V_out_* on 5 chips.

To verify the detection capability of the device, we first evaluated its temperature control ability. As shown in Figure 3a, it takes 4 min to heat up from 37 °C to 65 °C. The temperature fluctuation during 30 min of the LAMP reaction is controlled within 0.1 °C. Subsequently, through analyzing *V_out_* from 262,144 sensors, we tested solutions of different pH and verified that there is a linear relationship between pH and *V_out_*, while the sensitivity of the array’s response to H^+^ is 365.7 mV/pH (Figure 3b). To ensure the ISFETs’ selective response only to pH, the *V_out_* of MgSO_4_ (ranging from 2 mM to 10 mM) and KCl solutions (ranging from 25 mM to 125 mM) were also measured. According to Figure 3c,d, it can be seen that changing the concentration of MgSO_4_ and KCl, important components of the LAMP system, does not influence *V_out_* significantly. To validate the reusability and reproducibility of the detection platform, we conducted experimental verification from two dimensions: intra-chip and inter-chip consistency. As shown in Figure 3e, the results from the five repeated measurements on the same solution using a single chip showed no significant difference, demonstrating high repeatability across multiple tests. Furthermore, as illustrated in Figure 3f, the detection results from five chips showed no notable variations, indicating high inter-chip reproducibility.

### 3.2. LAMP Primer Design and Conservation Analysis

**Figure 4:**
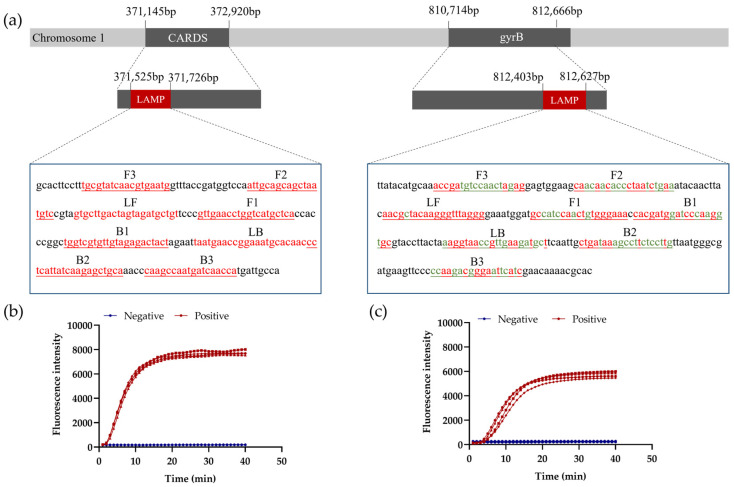
LAMP primer design and conservation analysis. (**a**) Position, length, and conservation analysis of primers. Red bases represent >99% conservation, and green bases are between 95% and 99% conserved. (**b**) Amplification curves of selected primer targeting *CARDS* gene; (**c**) amplification curves of selected primer against *gyrB* gene. Four duplicates were used for both negative and positive tests (added template is 4 × 10^3^ copies/mL).

The sequences of the MP *CARDS*, *gyrB*, and *SDC1* genes were all downloaded from the NCBI database, containing a total of 50 sequences for *CARDS*, 332 sequences for *gyrB*, and 3 sequences for *SDC1*. The multiple sequence comparison results showed that the *CARDS* and *gyrB* genes were more conservative. To cover most stains with different mutations, a combination of two primer sets targeting the conserved sequences of the *CARDS* and *gyrB* genes was designed. Initially, 11 and 10 sets of primers were designed for the *CARDS* and *gyrB* genes, respectively. Finally, one primer set of each target with the best time to positive (TTP) was selected (Figure 4b,c). Figure 4a illustrates the location and length of the target gene, as well as the conservation of each base of the selected primers. Primers targeting the *CARDS* gene are all >99% conserved, and primers for the *gyrB* gene are all >95% conserved at the base level, with some being >99%. This demonstrates that the selected primers are suitable for efficient and specific amplification.

### 3.3. Optimization and Performance of LAMP Assay

**Figure 5:**
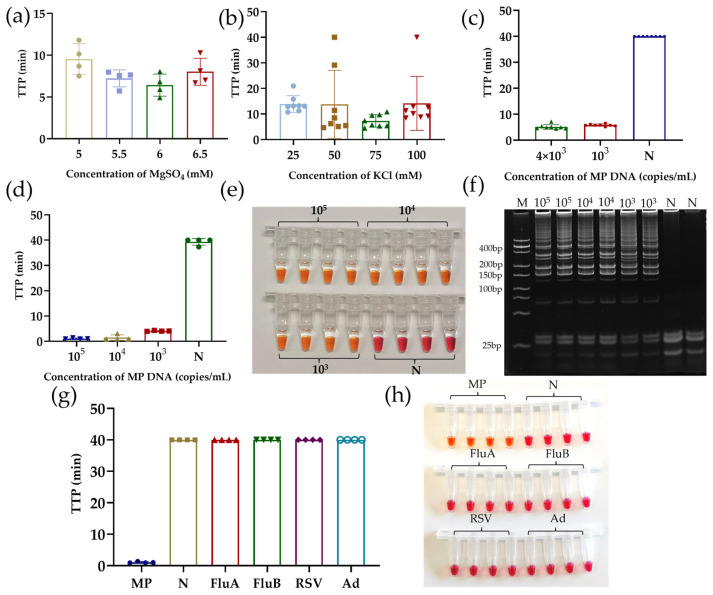
Optimization and performance of LAMP assay. (**a**) MgSO_4_ concentration gradient optimization using fluorescent LAMP; (**b**) KCl concentration gradient optimization using fluorescent LAMP; (**c**) sensitivity of fluorescent LAMP system; (**d**) sensitivity of low-buffered pH-LAMP; (**e**) end-point color change in pH-LAMP in (**d**); (**f**) electrophoresis bands of LAMP products from (**d**); (**g**) validation of pH-LAMP system for cross-reactivity with other respiratory pathogens; (**h**) end-point color change in cross-reaction validation in (**g**). Negative results were plotted at TTP = 40.

A gradient of MgSO_4_ and KCl concentrations of the system was optimized (shown in Figure 5a,b) to improve the amplification efficiency and specificity of the assay. The ideal TTP was obtained when MgSO_4_ is 5.5 mM and KCl is 75 mM. For the final fluorescent LAMP system, the results in Figure 5c demonstrate that 10^3^ copies/mL of MP DNA can be detected in less than 10 min. Meanwhile, there was no non-specific amplification in the negative system. Then, we converted the verified fluorescent LAMP system to a low-buffered pH-LAMP system for the use of the ISFET chips. The concentration of the pH buffer Tris-HCl was reduced while KOH was added for the initial pH adjustment (8.20 ± 0.01). This pH allows for efficient LAMP amplification. The detection results of the optimized low-buffered LAMP are shown in Figure 5d. The sensitivity of the low-buffered assay is still 10^3^ copies/mL. After the amplification, the system underwent an obvious color change from red to yellow (Figure 5e) with the use of a phenol red pH indicator as an internal control, indicating a significant pH change in the positive reaction. To determine the specificity of the amplification products, we analyzed the LAMP products by polyacrylamide gel electrophoresis. The distribution of the bands (Figure 5f) shows that all the products were specific at template concentrations from 10^5^ to 10^3^ copies/mL, while no non-specific products were generated in the negative group. For the specificity test, the addition of different respiratory pathogen templates (Figure 5g,h) shows no obvious pH change or color change. Therefore, the pH-LAMP system does not cross-react with common respiratory pathogens (FluA, FluB, MP, Ad).

### 3.4. MP Detection with ISFET Sensors with pH-LAMP

**Figure 6:**
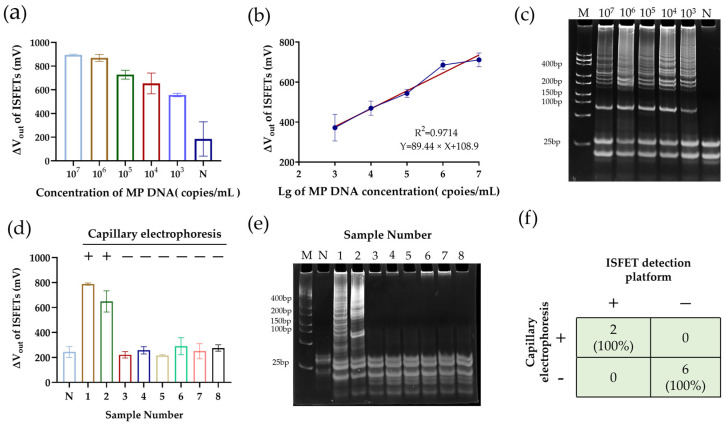
Performance of low-buffered pH-LAMP assay on ISFET array. (**a**) Values of changes in voltage signals after amplification of systems with different concentrations of MP template added. (**b**) *V_out_* difference between positive group and negative control in (**a**). (**c**) Electrophoretic bands of LAMP amplification products from (**a**). (**d**) Values of changes in voltage signals after amplification of 8 clinical throat swab samples, as well as clinical laboratory diagnostic results. (**e**) Electrophoretic bands of LAMP amplification products from (**d**). (**f**) Overall diagnostic performance of ISFET detection platform.

As shown in Figure 5, the off-chip optimized pH-LAMP system enables the specific and highly sensitive detection of MP. To determine the performance of the integrated ISFET pH-LAMP system, low-buffered pH-LAMP assays with gradient MP DNA (10^7^ to 10^3^ copies/mL) were performed on the ISFET array chip (Figure 2b), along with the negative control. After amplification at 65 °C for 30 min, the ISFET array output different voltage signals with the addition of varied concentrations of MP DNA from 10^7^ to 10^3^ copies/mL, as shown in Figure 6a. The Δ*V_out_* of the positive system was significantly higher than that of the negative control. Based on the experimental results, we observed that within the concentration range of 10*^3^* to 10^7^ copies/mL, the increase in voltage signals from the ISFET sensor exhibited a linear relationship with the initial concentration of the target DNA, as shown in Figure 6b (R^2^ = 0.9714). However, when the concentration reached 10^7^ copies/mL and above, the increase in voltage signals significantly slowed down, suggesting that the sensor may have reached its detection saturation state. Then, the on-chip amplified products were collected for polyacrylamide gel electrophoresis, and increased voltage signals were verified by the electrophoresis bands, as shown in Figure 6c. A combined analysis of the voltage signals and the electrophoretic bands showed that the sensitivity of this detection platform reached 10^3^ copies/mL (approximately 1 copy per reaction), comparable to the off-chip LAMP assay. To further verify the reliability of the ISFET detection system, we collected eight throat swab samples and tested them using both the ISFET sensors developed in this study and PCR-based capillary electrophoresis in clinical laboratory diagnosis. The detection results, as shown in Figure 6d,e, reveal that sample 1 and sample 2 are MP-positive, while the other samples are negative, fully consistent with the clinical laboratory diagnosis, confirming the accuracy and reliability of the ISFET system in detecting real-world samples.

## 4. Discussion

There are still obvious drawbacks to current diagnostic methods for MP infections, such as them being high-cost and time-consuming and having reliance on specialized laboratories, while patients who cannot be accurately diagnosed in a timely manner may receive unnecessary antibiotic therapy or risk misdiagnosis. Therefore, it is urgent to establish a rapid, low-cost, accurate, and portable miniaturized testing platform [28,29]. In response to such needs, our study provides an electronic label-free assay platform for MP, combining LAMP with an ISFET array.

The highly sensitive and specific LAMP system is one of the key components of the detection platform. Firstly, we screened the conserved genes of MP, including the *CARDS* and *gyrB* genes, and performed targeted primer design. The compositionally optimized low-buffered LAMP system does not cross-react with other respiratory pathogens. With a sensitivity up to 10^3^ copies/mL, the system undergoes a significant pH change at the end of amplification. There is a color change at the end, which can be judged by the naked eye. But the colorimetric method is somewhat subjective in this way. The by-product of the amplification, H^+^, responds sensitively and selectively, as detected by the ISFET sensor (365.7 mV/pH), producing a value which is much higher than that of Nernstian sensitivity (59.5 mV/pH) [27]. Furthermore, the detection results from this device demonstrate strong reproducibility and consistency, ensuring reliable and stable performance across repeated tests and different samples. Thus, the on-chip detection platform combining LAMP and ISFET sensors provides a feasible means for MP testing.

Of the ISFET biosensors reported by previous researchers, a smaller number of detection chip sensors were used, along with larger chip sizes and higher reagent requirements [30,31]. In contrast, the sensing area of the detection chip in this device consists of 512 × 512 ISFET arrays, but the diameter of the sensing pad is only 3 μm, and the reaction system required for a single chip is only 1 μL, which makes the cost of reagents lower. With 262,144 sensors performing simultaneous signal acquisition, a large number of test results greatly increases the fault tolerance of the platform and provides more accurate test results.

The effective combination of the low-buffered LAMP system, which is highly productive in H^+^ generation, and the ISFET array, which is highly sensitive to H^+^, enables the non-labeled, rapid, and accurate detection of MP. The *V_out_* of the positive group adding gradient concentrations of MP DNA (10^7^ to 10^3^ copies/mL) increased significantly at the end of amplification. Δ*V_out_* was statistically higher than that of the negative group, while the detection limit was about 10^3^ copies/mL (approximately 1 copy per reaction). Further analysis, comparing the voltage values of 10^7^ to 10^3^ copies/mL to the control group, revealed a linear relationship with the template concentration. This range covers a wide spectrum from low to high concentrations, making it suitable for most practical detection needs. To further verify the reliability of the ISFET detection system, we tested eight throat swab samples. And the entire process, from sample collection to result interpretation, takes about 60 min, significantly shorter than the time required for current clinical diagnostic methods. Additionally, the procedure is straightforward and does not require complex or expensive instruments.

In summary, our proposed detection device can be used to build a non-labeled and rapid electrical detection platform for the molecular detection of targets such as pathogens. Especially in relatively resource-poor settings and emergency care, etc., it enables the rapid screening and diagnosis of patients to accelerate clinical decision making and enable timely therapeutic and control interventions.

## 5. Conclusions

This paper presents the rapid electronic detection of MP DNA on an integrated platform. This platform uses a 512 × 512 CMOS-based ISFET array, which is highly sensitive (365.7 mV/pH) and specific to H^+^. Also, it demonstrates acceptable reproducibility and consistency. The low-buffered LAMP system targeting MP DNA is not capable of the amplification of other pathogens. And there is an obvious pH change at the end. The specific pH-LAMP system and highly sensitive bio-chip are combined to achieve the label-free detection of MP. The *V_out_* of the positive group is significantly higher than that of the negative control, and the voltage difference with the negative control is linearly related to MP concentration (R^2^ = 0.9714). The device achieves high sensitivity for MP detection (10^3^ copies/mL). The platform’s ability to rapidly and accurately detect clinical samples also confirms its significant application value in clinical detection. We built a label-free electrical detection platform for rapid molecular detection. And in resource-limited settings, this platform is expected to further validate the rapid diagnosis of infectious diseases at the point of care, epidemiological surveillance, and reduction in antimicrobial resistance.

## Data Availability

Data are contained within this article.
